# Genetic determinants of circulating haptoglobin concentration

**DOI:** 10.1016/j.cca.2019.03.1617

**Published:** 2019-07

**Authors:** Nabila Kazmi, Yoshiro Koda, Ndeye Coumba Ndiaye, Sophie Visvikis-Siest, Matthew J Morton, Tom R Gaunt, Ian Galea

**Affiliations:** a MRC Integrative Epidemiology Unit (IEU), Population Health Sciences, Bristol Medical School, University of Bristol, Bristol, UK; b Department of Forensic Medicine, Kurume University School of Medicine, Kurume 830-0011, Japan; c Université de Lorraine, Inserm, IGE-PCV, Nancy, France; d Clinical Neurosciences, Clinical & Experimental Sciences, Faculty of Medicine, University of Southampton, UK

**Keywords:** ALSPAC, Haptoglobin, Copy number variant, Hp, haptoglobin, Hb, haemoglobin, CNV, copy number variant, SNP, single nucleotide polymorphism, LD, linkage disequilibrium, GWAS, genome-wide array screening, ALSPAC, Avon Longitudinal Study of Parents and Children, SFS, STANISLAS Family Study, CI, confidence interval, HWE, Hardy-Weinberg equilibrium

## Abstract

Haptoglobin (Hp) is a major plasma acute-phase glycoprotein, which binds free haemoglobin to neutralize its toxicity. The *HP* gene exists as two copy number variants (CNV), Hp1 and HP2, which differ in two ways: serum Hp level and functional differences in Hp protein products. Both mechanisms may underlie the *HP* CNV's influence on susceptibility and/or outcome in several diseases. A single nucleotide polymorphism rs2000999 has also been associated with serum Hp level. In a meta-analysis of three studies from England, France and Japan, with a combined sample size of 1210 participants, we show that rs2000999's effect on circulating Hp level is independent from that of the *HP* CNV. The combined use of rs2000999 and the *HP* CNV can be an important genetic epidemiological tool to discriminate between the two potential mechanisms underlying differences between HP1 and HP2 alleles.

## Highlights

• Haptoglobin (Hp) is a major plasma protein, with multiple biological functions.
• HP1 and HP2 alleles differ in two ways: Hp expression level and biological function.
• The single nucleotide polymorphism rs2000999 is related to Hp expression level.
• In a sample size of 1210, we show that rs2000999 and *HP* allele status have independent effects on serum Hp level.
• The use of rs2000999 can inform about the mechanism of differences between HP1 and HP2 alleles.

## 1 Introduction

Haptoglobin (Hp) is a major plasma acute-phase glycoprotein. The main biological function of Hp is to bind free haemoglobin (Hb) to prevent the loss of iron and subsequent kidney damage following intravascular hemolysis [1]; it may also have immunomodulatory properties [1]. In humans, the *HP* locus is polymorphic. The basic structure of Hp is of an alpha and beta chain that form a monomer; a disulphide bond between the alpha chains of two monomers leads to the formation of a dimer in the mature form of Hp. An intragenic duplication of exons 3 and 4 of the ancestral *HP* gene produced the HP2 allele, after which recurrent deletions produced the modern HP1 allele [2]. Therefore individuals could be one of three genotypes: HP1–1, HP2–1 or HP2–2 [3]. This copy number variant (CNV) codes for an extra cysteine residue in the alpha chain that forms an additional disulphide bond; therefore trimeric, tetrameric and higher order polymers are formed in individuals carrying the HP2 allele [3].

The *HP* CNV may influence the susceptibility and/or outcome in several diseases, for example, diabetes mellitus [4,5], atherosclerosis [6] and cardiovascular disease [7], malignancies [8] and infections [9], with HP2 conferring higher risk. The HP2 allele is associated with a dose-dependent reduction in serum Hp level, compared to HP1 (serum Hp level: HP1–1 > HP2–1 > HP2–2) [10]. The HP1 and HP2 alleles differ in their ability to clear Hb and may have other functional differences relating to protection from Hb-induced oxidative stress [11]. Hence the differential effects of HP1 and HP2 alleles could be mediated by a difference in levels of circulating Hp, or functional differences in their protein products, or both.

A single nucleotide polymorphism (SNP) rs2000999 has also been associated with plasma Hp level, resulting in a dose-dependent variation across the genotypes: GG > GA > AA [12]. Since rs2000999 is downstream of the *HP* gene, its effect is probably mediated via linkage disequilibrium (LD) with upstream variations, such as the single base-pair deletion rs35283911 [10]. Up to 45% of the variance in circulating Hp level is explained by rs2000999 [12]. It is likely that some of the effects of rs2000999 and the *HP* CNV on Hp level are independent of each other [2,10,13].

The aim of this study was to perform a meta-analysis to confirm the independence of rs2000999 from the *HP* CNV with respect to circulating Hp level. If rs2000999 truly has an independent effect on Hp level, the combined use of rs2000999 and the *HP* CNV can be an important genetic epidemiological tool to discriminate between the two potential mechanisms underlying differences between HP1 and HP2 alleles: Hp protein function or Hp protein expression level.

## 2 Material and methods

### 2.1 Participants

We included studies which: (1) genotyped both the *HP* CNV and rs2000999 and (2) genotyped the CNV using gold-standard PCR approaches (Table 1), rather than imputation from genome-wide array screening (GWAS). Three studies were identified at the time of study planning, and data was made available by the authors. The Avon Longitudinal Study of Parents and Children (ALSPAC) is a general population cohort study [14,15]; lithium heparin plasma Hp level and *HP* genetic data was available from 325 participants (159 females and 166 males, Caucasian, age 18 ± 0.33 years). The ALSPAC study website (http://www.bristol.ac.uk/alspac/researchers/our-data/) contains details of all the data that is available through a fully searchable data dictionary and variable search tool. Ethical approval for the study was obtained from the ALSPAC Ethics and Law Committee and the Local Research Ethics Committees. The STANISLAS Family Study (SFS) provided data from 500 French community-dwelling adult women of European ancestry (mean age 39.6 ± 4.1 years) [12,16]. Soejima et al consisted of 385 Japanese patients scheduled for blood transfusion (195 females and 190 males, Asian, adults of undetermined age) [13]. Table 1 lists the study characteristics including the methods used for *HP* genotyping and circulating Hp level assay.

**Table 1:** Study populations.

Study	N	Gender (% female)	Mean age (± SD)	Country	Source	*HP* CNV	rs2000999	Hp assay	Mean Hp level (± SD)	Ref
ALSPAC	325	49%	18 ± 0.33	England	Community	ARCS^b^	GWSA	Immuno-turbimetry on plasma^e^	0.99 ± 0.47	Fraser et al. [14,15]
SFS	500	100%	39.6 ± 4.1	France	Community	PCR^c^	GWSA	Immuno-nephelometry on plasma [12]	1.07 ± 0.43	Visvikis-Siest S et al. [16]
Japan	385	51%	Adult^a^	Japan	Hospital	qPCR^d^	qPCR	ELISA on serum [13]	1.42 ± 1.07	Soejima et al. [13]

ARCS = amplification ratio control system, qPCR = quantitative polymerase chain reaction, GWSA = genome-wide SNP array, ELISA = enzyme-linked immunosorbent assay.

a Adult, exact age undetermined.

b doi:https://doi.org/10.1093/nar/gkr046.

c http://clinchem.aaccjnls.org/content/48/9/1377.

d doi:https://doi.org/10.1373/clinchem.2008.113126.

e https://usdiagnostics.roche.com/products/03005593322/PARAM182/overlay.html

### 2.2 Analysis

The contribution of the *HP* CNV and rs2000999 to circulating Hp levels was studied within each cohort using multivariate regression modelling in R (Hp level as outcome, *HP* CNV as exposure and rs2000999 as covariate). The interaction terms *HP* CNV * gender and rs2000999 * gender were considered, except in the female-only French cohort. The results of each cohort were combined in a fixed-effects meta-analysis conducted using the R package *metaphor*, with the inverse variance for weighting and the restricted maximum likelihood estimator method as heterogeneity estimator, and assuming a standard normal distribution to calculate confidence intervals (CI).

## 3 Results

### 3.1 Contribution of *HP* polymorphism to expression

Data from the two mixed gender British and Japanese cohorts and the female-only French cohort was used to assess the contribution of the two *HP* genetic polymorphisms to circulating Hp level. All cohorts were in Hardy-Weinberg equilibrium (HWE) for both genetic variants (Supplementary File 1, Table S1). We found evidence of LD between the two polymorphisms in each cohort (Supplementary File 1, Table S1).

**Fig. 1:**
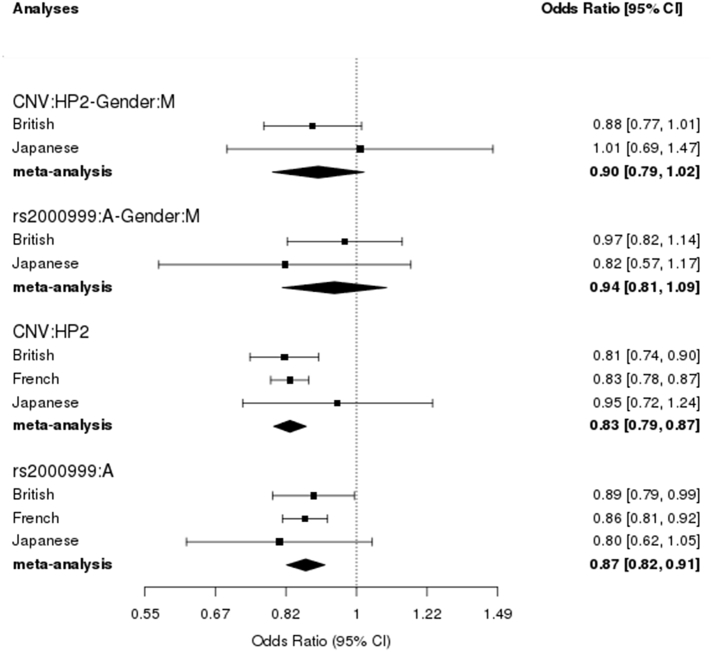
Effect of the *HP*CNV and rs2000999 on circulating Hp level in multivariate analyses. CNV was included in the model as a dependent variable and rs2000999 as a covariate whereas gender was included as an interaction term. The labels indicate the comparison being made; for instance CNV:HP2 indicates the change in Hp level in g/L for every HP2 allele (i.e. the beta coefficient). Gender:M = the gender is male.

**Fig. 2:**
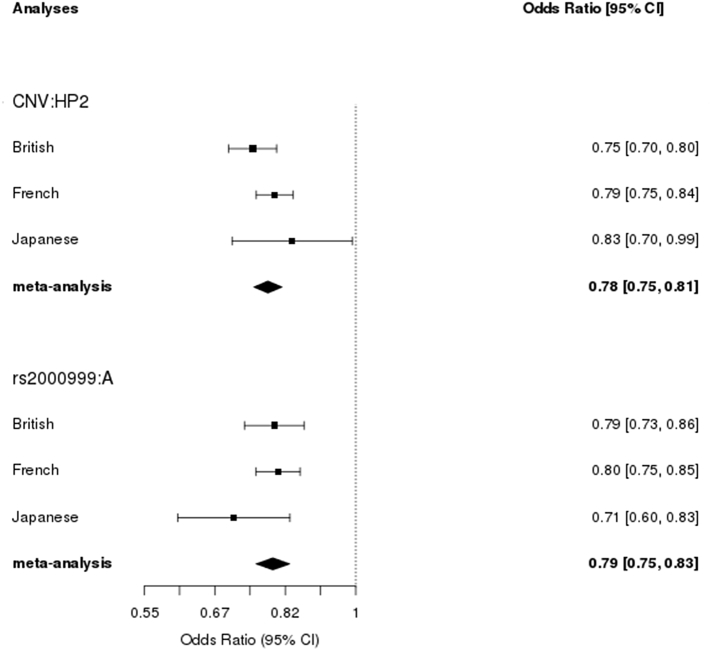
Effect of *HP*-CNV and rs2000999 on circulating Hp level in univariate analyses.

To determine the contribution of *HP* genetic variation to circulating Hp level we performed multivariate linear regression where *HP* CNV was the exposure and rs2000999 was the covariate and combined the effects of each cohort in a fixed-effects meta-analysis. The analysis identified that the rs2000999 minor allele (A) was associated with a Hp change of −0.14 g/L (95% CI −0.20 to −0.09, *p* = 2.6 × 10^−7^), and the *HP* CNV was also associated with Hp level (for each HP2 allele, β = −0.189 g/L, 95% CI −0.24 to −0.14, *p* = 1.68 × 10^−15^), Fig. 1. There was no statistical evidence of between study heterogeneity (Cochrane Q *p*-values were > 0.4). The magnitude of effect was larger in univariate compared to multivariate analysis (cf Fig. 1, Fig. 2). The French and British studies, which restricted participants to healthy individuals only, broadly revealed the same results (Fig. 1, Fig. 2).

The main effect of gender was assessed in British and Japanese cohorts (British: rs2000999 *p* = .69, *HP* CNV *p* = .08; Japanese: rs2000999 *p* = .10, *HP* CNV *p* = .96). There was no interaction of gender with either the *HP* CNV or rs2000999 in determining Hp level (for meta-analysis: rs2000999 *p* = .4 and *HP* CNV p = .1, Fig. 2).

The mean Hp level was 0.99 g/L (standard deviation (SD): 047) in the British cohort, 1.42 g/L (SD: 1.06) in the Japanese cohort and 1.06 g/L (SD: 0.43) in the French cohort. *HP2* and rs2000999:A were more frequent in Japanese versus Caucasian populations (χ^2^ = 27.04 and 71.80 respectively, degrees of freedom = 2 and 2 respectively, *p* < .00001 in both cases). In a multivariate linear regression, the effect of ancestry (Asian/European) on Hp level persisted despite controlling for the *HP* CNV, rs2000999 and gender (Supplementary File 1, Table S2).

## 4 Discussion

This meta-analysis of *HP* genetic variation and circulating Hp concentration across British, Japanese and French populations showed that: (1) both rs2000999 and *HP* CNV contribute to Hp level, (2) the effects of rs2000999 and the *HP* CNV are independent of each other, i.e. LD does not fully account for their combined effects on Hp level. The beta coefficients of univariate regression analyses were consistently higher than the respective multivariate analyses indicating that LD accounts for some of the effect seen in univariate analyses, but clear and strong independent effects were observed.

Other factors potentially influencing circulating Hp level include age, gender and ancestry. We could not include age as a covariate since participants in the British study were roughly of the same age, and the Japanese study did not collect age data. However, in the French cohort, age above 18 years did not influence Hp level [12,17]. Gender may affect circulating Hp level [18], so it was included as a covariate, but it did not have a significant relationship to Hp level in our analyses. Ancestry also impacts on circulating Hp level [13]. Some of the effect of ancestry could be due to inter-population differences in the frequencies of the *HP* CNV and rs2000999, but we show here that the effect of Asian/European status on Hp level persisted despite controlling for the *HP* CNV, rs2000999 and gender. This residual effect of ancestry could be due to other genetic determinants. However, comparison of studies performed in different populations needs to be interpreted with caution, since differences in Hp level could also be due to methodological differences in Hp assay technique between studies.

Strengths of this study include the large sample size and the multivariate nature of the analysis, controlling for gender, ancestry, *HP* CNV and *HP* SNP. Two of the populations (French and British) were unselected individuals allowing us to perform sensitivity analysis without the confounding factor of disease that exists in the Japanese study. Also the *HP* CNV was genotyped using a gold-standard PCR approach, not determined by imputation.

This study has a number of limitations. Other covariates potentially affecting Hp level, such as body mass index and cigarette consumption [17], were not available. The Japanese cohort was not community-based since it was a population of patients requiring blood transfusion. This could have been responsible for the observed higher Japanese frequency of HP2 and rs2000999:A, despite the population being in HWE. It is still possible that some of the higher Hp level in the Japanese cohort, unexplained by the *HP* CNV and rs2000999, was driven by underlying medical conditions in people requiring blood transfusion, as well as other population-specific genetic determinants.

## 5 Conclusions

In conclusion, we show that the *HP* CNV and SNP both contribute to circulating Hp level, independent of each other. Using these two *HP* genetic variations together is a valuable genetic epidemiological tool to dissect the mechanism underlying the differential effect of HP1 and HP2 alleles in disease, namely whether this is through Hp level or differences in function between Hp polymers.

## Contributors

Concept: TG, IG. Data contributors: NK, YK, NCN, SVS, TG. Analysis: NK, MM, IG. Manuscript: all authors.

## Funding statement

The UK Medical Research Council (MRC) and Wellcome Trust (Grant ref.: 102215/2/13/2) and the University of Bristol provide core support for ALSPAC. This publication is the work of the authors and TG, IG and NK are guarantors. A comprehensive list of grant funding is available on the ALSPAC website (http://www.bristol.ac.uk/alspac/external/documents/grant-acknowledgements.pdf). This research was funded by UK MRC grant MR/L01453X/1 (MM, IG) and by Cancer Research UK program grant C18281/A19169 (NK). Tom Gaunt receives funding from the UK MRC (MRC Integrative Epidemiology Unit, MC_UU_00011/4). ALSPAC GWAS data was generated by Sample Logistics and Genotyping Facilities at Wellcome Sanger Institute and LabCorp (Laboratory Corporation of America) using support from 23andMe. A comprehensive list of grant funding is available on the ALSPAC website (http://www.bristol.ac.uk/alspac/external/documents/grant-acknowledgements.pdf).

## Conflict of interest

The authors declare no conflict of interest.

## Appendix A Supplementary data

Additional analyses. Image 1
